# Trends in endometriosis hospitalizations in Germany, 2000-2023

**DOI:** 10.1186/s12905-026-04306-4

**Published:** 2026-02-13

**Authors:** Valeria Schellenberg, Till Neugebauer, Yüce Yilmaz-Aslan, Patrick Brzoska

**Affiliations:** Health Services Research, Faculty of Health, School of Medicine, Witten/Herdecke University, Alfred-Herrhausen-Straße 50, 58448 Witten, Germany

**Keywords:** Endometriosis, Hospitalizations, Inpatient care, Germany

## Abstract

**Background:**

Endometriosis is the second most common gynecological condition among women of reproductive age. The most common symptoms include severe lower abdominal pain, heavy and irregular menstrual bleeding, and impaired fertility. Both diagnosis and treatment are often associated with surgical procedures. Therefore, patients with endometriosis still require inpatient care more frequently than women with other conditions. Understanding hospitalization trends can shed light on disease diagnosis, treatment, and management, as well as regional disparities in access to care. The present study examines hospitalization trends for endometriosis in Germany, focusing on regional differences at the federal state level.

**Methods:**

Based on data from the German Federal Statistical Office, we analyzed annual hospitalizations for endometriosis (ICD-10 N80), for the period 2000 to 2023. Age-standardized hospitalization rates (ASHRs) per 100,000 women, using the 2013 European standard population, were examined. We applied joinpoint regression analysis to identify temporal trends and calculated Average Annual Percent Changes (AAPCs) with 95% confidence intervals. Descriptive statistics and choropleth maps were used to visualize regional variation, long-term trends, and relative changes in ASHRs between 2000 and 2023.

**Results:**

All 16 federal states recorded increases in ASHRs over the 24-year period. In 2000, the national rate was 43.7 per 100,000 women, rising to 88.1 per 100,000 in 2023. Substantial regional differences in endometriosis hospitalization rates were observed across Germany’s federal states. For 2023, the highest rate was recorded in North Rhine-Westphalia with 112.4 hospitalizations per 100,000 women, followed closely by Saarland (103.1). In contrast, the lowest rates were seen in Hamburg (43.2) and Bremen (53.1). Also, increases in ASHRs over the study period varied considerably between states, with the largest increases observed in Berlin, Bavaria and Brandenburg (+ 196.2%, + 173.2% and + 165.6%, respectively) and the lowest observed in Bremen, Lower Saxony, Hamburg and Saarland (+ 36.5%, + 41.1%, + 52.1% and + 51.2%, respectively).

**Conclusions:**

The spatial patterns highlight considerable regional heterogeneity in the development of endometriosis-related hospitalizations over the past two decades and suggest the influence of structural, demographic, or healthcare system factors at the state level. Limited data hinders a comprehensive understanding of these differences, emphasizing the need for mixed-methods research.

## Introduction

Endometriosis is a chronic, estrogen-dependent inflammatory condition and ranks as the second most common benign gynecological disease among women of reproductive age [1]. It is characterized by the growth of tissue resembling the endometrium outside the uterine cavity, most frequently involving the ovaries, fallopian tubes, and adjacent pelvic structures [2, 3]. In most cases endometriotic lesions are found in the abdominal and pelvic cavities, on the ovaries, intestines or peritoneum. However, endometriotic tissue has the capacity to grow in any part of the body and in rare cases may even occur outside the abdominal cavity, such as in the lungs [3, 4].

This misplaced tissue responds to hormonal cycles and can lead to chronic pain and a significantly reduced quality of life. Symptoms typically occur in connection with the menstrual cycle but can develop over time into chronic pain that can affect different parts of the body [5, 6]. Common symptoms typically include pelvic pain, infertility, dysmenorrhea, deep dyspareunia, dysuria, dyschezia, and fatigue which together affect a person’s physical, mental, and sexual health as well as their social well-being [3, 7, 8]. In addition, endometriosis is frequently associated with several comorbidities, such as irritable bowel syndrome, interstitial cystitis/bladder pain syndrome, depression and anxiety disorders [8–10].

Endometriosis affects an estimated 10% of girls and women, primarily those aged between 15 and 49 years [11]. According to the World Health Organization, approximately 190 million individuals worldwide are currently living with the disease. In Germany, around two million women are affected with up to 40,000 new cases reported each year [1]. However, accurately determining the true prevalence and incidence of endometriosis remains challenging [12]. As a result, experts assume that there is a considerable number of unreported cases, which further complicates early detection and access to adequate care [1]. It is particularly problematic that a definitive diagnosis, as classified under the International Statistical Classification of Diseases and Related Health Problems (ICD-10-GM, code N80), still requires surgical visualization of the endometriotic lesions, usually confirmed by histological examination [4, 13]. According to the S2k guideline for endometriosis, published by the German Society of Gynecology and Obstetrics (DGGG), this is usually done by laparoscopy, during which tissue samples are obtained [4]. Laparoscopy is considered the international standard for diagnosis, as it not only confirms the presence of endometriosis but also allows for detailed assessment of the location, extent, and type of lesions and cysts [14, 15].

Moreover, laparoscopies often serve as the initial therapeutic intervention, enabling the removal or ablation of endometriotic tissue during the same procedure [16]. The primary goal of surgical intervention is the complete removal of endometriotic lesions in order to reduce the local inflammatory response and to restore anatomical structures as fully as possible [4, 17]. However, due to the chronic nature of endometriosis, the disease has a high recurrence rate even after surgical treatment [17]. For this reason, depending on the patient’s age, symptoms, and individual life circumstances, more radical surgical approaches may become necessary in certain cases. These include hysterectomy (surgical removal of the uterus) and adnexectomy (removal of the fallopian tubes and/or ovaries) [19, 20].

Therefore, patients with endometriosis still require inpatient care more frequently than women with other conditions, both for diagnostic and therapeutic reasons [20]. In Germany, in accordance with the S2k guidelines, both laparoscopy and surgical treatment of endometriosis are typically performed in certified endometriosis centers (*Endometriosezentren*). These are specialized hospital units with multidisciplinary expertise, ensuring standardized diagnostics, surgical management, and follow-up care according to current evidence-based recommendations [4]. Despite specialized care structures, the new clinical guidelines and increasing social awareness of the disease, which heightened sensitivity among both patients and physicians, it remains unclear how these developments have translated into healthcare utilization over time [4, 22]. This applies in particular to the inpatient sector, where the impact of these changes have not yet been fully understood.

A deeper understanding of hospitalization patterns could reveal how the disease is currently being identified, treated, and managed within the healthcare system and how these aspects have evolved over time. Analyzing hospital admission trends may thus provide valuable insights into the burden of disease, diagnostic activity, and structural inequalities in access to care across different regions. Yet, detailed data on the current healthcare situation of endometriosis patients, as well as on the temporal development of hospitalizations related to the disease in Germany, remain scarce. Therefore, the aim of the present study was to analyze the trends in hospitalizations associated with endometriosis from 2000 to 2023. Particular attention was paid to capturing regional differences and the temporal dynamics at the federal state level.

## Methods

### Data sources

This study utilized data from the German Federal Statistical Office *(Statistisches Bundesamt)*, which systematically compiles hospital discharge records across the entire country. Data was obtained through the online database of Federal Health Reporting (www.gbe-bund.de). Specifically, we analyzed annual counts of hospitalizations for endometriosis, identified using the ICD-10 diagnosis code N80, for the period 2000 to 2023. The dataset comprises aggregated hospital discharge statistics (in the form of absolute numbers of discharges as well as crude and age-standardized rates of discharges per 100,000 female inhabitants) by year, age (5-year age groups) and region of residence and is publicly available, ensuring comprehensive national coverage and consistency in data reporting across all 16 German federal states *(Bundesländer)*^1^.

### Case definition

Hospitalization events were included if they recorded an ICD-10 code N80 for endometriosis as the discharge diagnosis. Because the data are aggregated and anonymized, repeated hospitalizations of the same individual could not be identified but are rather included as separated cases. Consequently, all results refer to hospitalization events and not unique patients. This limitation is particularly relevant in the context of endometriosis, which may involve recurring or chronic symptoms and repeated hospital contacts [14].

### Regional classification

Germany consists of 16 federal states, which served as the unit of regional comparison. We used age-standardized hospitalization rates (ASHRs) for each federal state and for the national average. This approach allows comparison of endometriosis hospitalization trends while adjusting for population structure differences across regions and over time.

### Statistical analysis

We drew on available age-standardized hospitalization rates per 100,000 women using the direct standardization method, employing the 2013 European population as the standard. These rates were retrieved for each year between 2000 and 2023 for all federal states and the national level.

To examine temporal trends, we conducted joinpoint regression analysis, a statistical technique that identifies years where significant changes in trends occur (called joinpoints). This analysis was performed using the Joinpoint Regression Program (Version 5.3.0) developed by the U.S. National Cancer Institute. Average Annual Percent Change (AAPC) estimates were calculated for each state, and 95% confidence intervals (CIs) were generated using the empirical quantile method. AAPC values provide a summary measure of long-term change in hospitalization rates over the study period.

Descriptive statistics, tables, and visualizations were generated using Gnumeric 1.12.17. Regional differences in hospitalization rates were further evaluated by calculating percentage changes between 2000 and 2023.

To visually illustrate regional differences and temporal trends in endometriosis-related hospitalizations, we created a series of choropleth maps. These maps display the age-standardized hospitalization rates for 2023, the Average Annual Percent Change (AAPC) for the period from 2000 to 2023, and the relative changes in hospitalization rates between 2000 and 2023. The maps were constructed using geographic data at the federal state level and allow for rapid identification of spatial patterns, such as regional clusters of high or low burden, or areas with especially steep increases over time. These visual tools support a better understanding of heterogeneity in disease dynamics and healthcare utilization across Germany and help identify regions that may require targeted public health interventions or further investigation. Choropleth maps were created using the Python library GeoPandas 1.1.1.

## Results

### National trends in hospitalizations for endometriosis

**Fig. 1:**
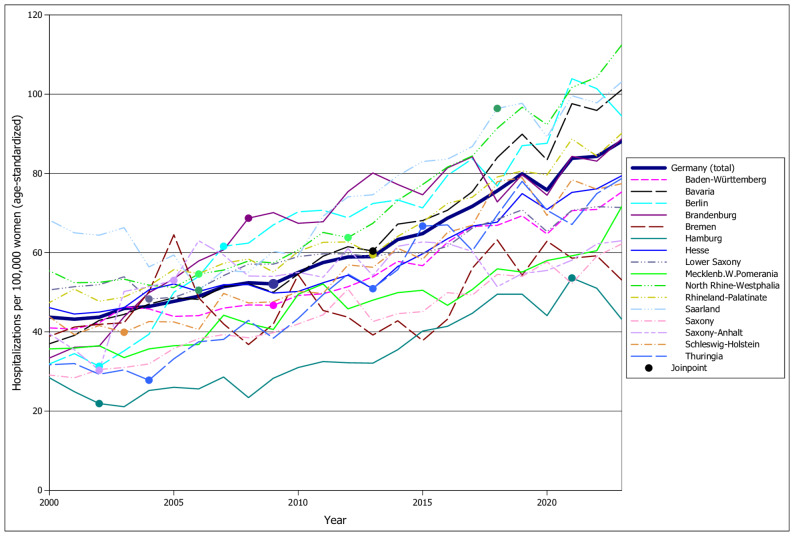
Endometriosis hospitalizations per 100,000 women (age-standardized) in Germany by federal state, 2000-2023

Between 2000 and 2023, the age-standardized hospitalization rate for endometriosis in Germany increased markedly. In 2000, the national rate was 43.7 per 100,000 women, rising to 88.1 per 100,000 in 2023. This represents a 101.6% increase over the 24-year period and corresponds to an Average Annual Percent Change (AAPC) of + 3.2% (Table 1). The trend was consistently upward, although one joinpoint was identified in the national series, suggesting a period of acceleration in rate starting in 2009 (Fig. 1).

**Table 1:** Endometriosis hospitalizations per 100,000 women (age-standardized) in Germany by federal state, 2000-2023

Region/federal state											Year														Change 2000 -2023 (%)	Number of joinpoints	AAPC	95%	-CI
	2023	2022	2021	2020	2019	2018	2017	2016	2015	2014	2013	2012	2011	2010	2009	2008	2007	2006	2005	2004	2003	2002	2001	2000					
Germany (total)	88.1	84.3	83.8	75.8	79.9	75.6	71.7	68.7	64.8	63.3	59.0	58.9	57.5	55.0	52.1	52.4	51.4	49.0	47.7	46.4	45.9	43.7	43.2	43.7	101.6	1	3.2	(3.04,	3.44)
Baden-Wuerttemberg	75.3	70.9	70.6	64.7	69.3	66.9	66.6	62.6	56.7	57.8	53.9	51.4	49.6	49.2	46.7	46.8	46.0	44.1	43.9	45.8	46.3	42.3	40.7	41.0	83.7	1	2.7	(2.36,	3.02)
Bavaria	101.1	95.9	97.6	83.4	89.9	84.0	75.4	70.7	68.1	67.2	60.4	61.5	59.1	54.6	50.0	52.1	51.4	48.2	48.6	47.0	44.4	42.9	39.1	37.0	173.2	1	4.3	(3.92,	4.73)
Berlin	94.5	101.4	103.9	87.6	87.0	76.8	83.7	79.5	71.3	73.3	72.4	68.8	70.7	70.3	67.0	62.4	61.6	54.0	50.0	39.4	35.2	31.3	34.5	31.9	196.2	2	4.8	(4.36,	5.53)
Brandenburg	88.7	83.1	84.3	74.5	79.7	72.8	84.2	81.4	74.6	77.2	80.1	75.4	67.8	67.4	70.1	68.7	60.7	57.9	53.4	49.9	43.8	36.4	36.1	33.4	165.6	1	4.2	(3.85,	4.64)
Bremen	53.1	59.2	58.6	62.9	54.3	63.2	56.1	43.2	37.8	42.8	39.2	43.7	45.4	54.4	42.0	36.8	42.0	48.6	64.5	50.1	42.3	41.9	41.2	38.9	36.5	0	1.3	(0.22,	2.39)
Hamburg	43.2	51.0	53.6	44.1	49.5	49.5	44.7	41.4	40.2	35.5	32.1	32.2	32.5	31.0	28.3	23.4	28.6	25.6	26.0	25.2	21.1	21.9	24.9	28.4	52.1	2	1.9	(1.03,	3.09)
Hesse	79.4	76.1	75.2	70.9	74.9	67.7	66.7	63.5	59.7	56.5	50.9	54.3	52.4	50.2	49.8	51.9	51.9	50.2	52.1	50.6	46.1	45.0	44.5	46.1	72.2	1	2.6	(2.12,	3.00)
Lower Saxony	71.4	71.6	70.7	65.3	70.8	68.4	66.2	61.6	59.8	60.2	58.9	59.7	59.7	59.0	57.1	57.1	54.2	50.9	48.7	48.3	53.9	51.9	51.4	50.6	41.1	1	1.5	(1.15,	2.00)
Mecklenb.-W.Pomerania	71.6	60.5	59.2	58.0	55.1	55.9	50.7	46.7	50.5	49.9	48.0	45.8	52.1	49.5	40.6	42.1	44.2	36.8	36.5	35.7	33.5	36.5	35.9	35.7	100.6	0	2.8	(2.30,	3.30)
North Rhine-Westphalia	112.4	104.3	101.7	92.3	96.7	91.4	84.4	81.6	77.2	73.2	67.4	63.8	65.1	60.7	57.4	57.9	55.6	54.6	51.2	51.8	53.3	52.5	52.4	55.4	102.9	2	3.2	(2.96,	3.51)
Rhineland-Palatinate	90.1	84.4	88.7	79.6	80.6	79.1	74.0	72.4	67.7	64.1	59.5	62.7	62.6	60.3	55.1	58.4	57.3	54.7	55.7	51.6	48.8	47.7	50.8	47.4	90.1	1	2.9	(2.49,	3.21)
Saarland	103.1	97.8	99.6	89.2	97.7	96.4	86.8	83.6	83.0	79.4	74.6	74.1	69.2	59.3	60.3	55.1	55.5	50.5	59.4	56.4	66.3	64.4	65.0	68.2	51.2	2	1.6	(1.04,	2.02)
Saxony	62.2	59.1	52.2	57.7	54.0	54.5	49.3	49.9	45.1	44.6	42.6	50.8	44.3	42.0	39.8	38.5	39.3	38.3	35.8	31.9	31.0	30.5	28.4	29.1	113.7	0	3.3	(2.86,	3.66)
Saxony-Anhalt	63.0	62.2	58.1	55.5	54.7	51.4	60.3	62.3	62.7	61.6	54.2	61.0	53.8	55.0	54.0	54.1	59.4	63.0	53.0	51.4	50.2	30.3	35.5	39.5	59.5	2	1.8	(0.88,	3.33)
Schleswig-Holstein	77.4	76.0	78.4	69.4	78.9	77.9	66.9	65.1	58.3	61.1	56.3	56.9	49.7	50.2	47.6	47.3	49.7	40.6	42.5	42.6	39.9	41.6	39.3	43.8	76.7	1	2.9	(2.41,	3.67)
Thuringia	78.7	74.9	67.1	70.7	78.0	69.5	60.5	67.0	66.7	55.7	51.0	54.6	49.4	43.4	38.4	42.9	38.1	37.5	33.2	27.8	30.4	29.3	32.0	31.7	148.3	2	4.0	(3.16,	4.71)

*AAPC *Average annual percentage change, *95%-CI *95% confidence interval

### Regional variation in 2023

Substantial regional differences in endometriosis hospitalization rates were observed across Germany’s federal states in 2023. The highest rate was recorded in North Rhine-Westphalia, with 112.4 hospitalizations per 100,000 women, followed closely by Saarland (103.1) and Bavaria (101.1). In contrast, the lowest rates were seen in Hamburg (43.2), Bremen (53.1), and Saxony (62.2). These figures reflect a 2.6-fold difference between the highest and lowest rates (Table 1).

### Regional trends from 2000 to 2023

Trends over time varied significantly between states (Table 1; Fig. 1). All 16 federal states recorded increases in age-standardized endometriosis hospitalization rates over the 24-year period. However, the rate of increase differed substantially, as shown by the AAPC values: The most pronounced increases were found in Berlin (AAPC = + 4.8%), Bavaria (+ 4.3%), and Brandenburg (+ 4.2%). In contrast, Bremen, Lower Saxony, Saarland, Saxony-Anhalt and Hamburg exhibited more moderate increases, with AAPCs below + 2.0%.

Joinpoint regression identified non-linear temporal patterns in several states. For instance, Berlin had two joinpoints, indicating periods of rapid increase (2002–2007) interspersed with decline (2000–2002) or slower growth (2007–2023). Other states, such as Saxony, exhibited stable upward trends without joinpoints, suggesting a consistent year-on-year increase (Fig. 1).

In some cases, the multiplicative change in hospitalization rates was striking: Bavaria’s rate grew by a factor of 2.73, while Berlin’s rate nearly tripled (factor 2.96) from 2000 to 2023. These differences emphasize not only varying absolute burdens but also distinct temporal trajectories across the country.

**Fig. 2:**
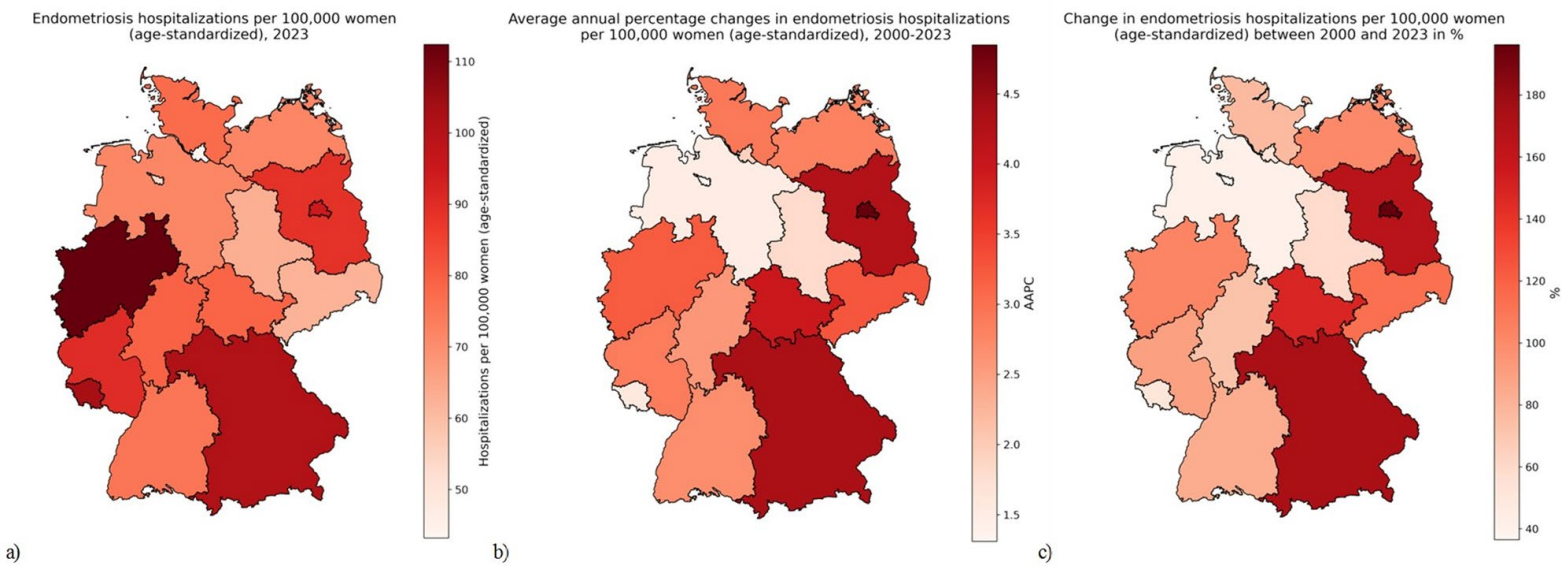
**a**-**c** Geographical distribution of endometriosis hospitalizations in Germany by federal state (**a**: hospitalizations per 100,000 women, 2023; **b**: Average annual percentage changes, 2000-2023; **c**: Change in hospitalizations between 2000 and 2023 in %)

To complement the statistical analyses, we generated three choropleth maps depicting regional differences in endometriosis-related hospitalization rates and temporal trends across Germany’s 16 federal states (Fig. 2a-c). The first map illustrates the age-standardized hospitalization rates (ASHR) for the year 2023, revealing marked geographic variation, with higher rates being observed in the Western and Southern parts of the country. This suggests potential disparities in disease burden, diagnostic practices, or access to care. The second map presents the average annual percent change (AAPC) in ASHRs between 2000 and 2023, while the third map show change in ASHRs. For both indices, some Eastern states and Bavaria showed moderate to strong increases, while Western states, displayed more stable or modestly increasing trends. These spatial patterns underscore considerable regional heterogeneity in the development of endometriosis-related hospitalizations over the past two decades and suggest the influence of structural, demographic, or healthcare system factors at the state level.

## Discussion

This study provides a comprehensive analysis of temporal and regional trends in hospitalizations associated with endometriosis in Germany over a 24-year period. The findings reveal a significant increase in age-standardized hospitalization rates at the national level and substantial variation across federal states, both in terms of level and trend dynamics.

### Interpretation of national trends

The age-standardized hospitalization rate for endometriosis in Germany more than doubled between 2000 and 2023, reaching 88.1 hospitalizations per 100,000 women in 2023. The increasing rate of inpatient stays is in line with national reference values. Previous German studies on endometriosis incidence based on administrative data align with the results of our analysis. For instance, a study by Kohring et al. (2024) observed a continuous rise in the incidence of diagnosed endometriosis between 2014 and 2022 [22]. Furthermore, a second analysis by the same authors reported a 65% increase in nationwide crude prevalence between 2012 and 2022 [13]. These results are consistent with the findings of our long-term trend analysis, which revealed a significant increase in the hospitalization rate for endometriosis in Germany. The marked rise in diagnoses may largely contribute to the observed increase in hospital admissions. However, the growth in inpatient cases does not necessarily reflect a higher disease prevalence [15]; rather, it likely results from improved diagnostic capabilities and/or increased utilization of inpatient care for endometriosis.

Several factors on the system-level, demographic, and sociocultural factors may help explain this development. It is likely that the significant increase in public and clinical awareness of endometriosis and its symptoms over the past two decades— both among patients and healthcare professionals —has led to more frequent diagnoses and increased referrals to specialist care [4, 13]. In particular, the Endometriosis Association Germany *(Endometriose-Vereinigung Deutschland e. V.)* has played a key role by providing information and support to affected individuals and by engaging in sustained health policy advocacy [21]. Moreover, advances in diagnostic methods and the dissemination of updated clinical guidelines [4] may have facilitated earlier diagnosis and more accurate coding of the disease. The expansion of specialized care structures and the growing density of endometriosis centers have further strengthened diagnostic capacity and access to treatment [22]. The currently high case numbers of endometriosis may, at least in part, be also attributable to the historically grown delay in diagnosing the condition [15]. Increased awareness in medical professionals as well as affected women, improved diagnostic methods, and earlier detection likely contribute to a rise in the number of women receiving inpatient treatment. Only in recent years has greater public attention been drawn to the issue through enhanced awareness campaigns and educational efforts [23]. As public awareness continues to grow, a further increase in diagnosed cases can be expected. Consequently, the number of hospitalizations may also rise, as more patients gain access to specialized care and undergo more intensive therapeutic interventions.

In contrast to our study, hospitalization rates for endometriosis have declined in countries such as France, Spain and the United States [4, 18, 25]. This trend may reflect changes in care pathways, including a greater use of outpatient management for mild to moderate cases and shifts in admission and treatment thresholds. In the German context, the persistently high and increasing hospitalization rates may therefore point to differences in care organization, referral patterns, and the timing of diagnosis and treatment, rather than to a simple lack of outpatient diagnostic capacity [4, 18, 25]. At the same time, the US study by Estes et al. (2019) [24] highlighted that an increase in surgical complications contributes to the burden on hospitals. Furthermore, the proportion of patients with multiple comorbidities has increased, as has the share of inpatient endometriosis admissions involving more complex cases [24]. When interpreted in light of these findings, the persistently high and increasing hospitalization rates observed in Germany may plausibly reflect, at least in part, a shift in inpatient case mix toward more complex clinical presentations. This interpretation is necessarily indirect and should be understood as hypothesis-generating rather than as a definitive conclusion, given that our data do not include information on disease severity or individual patient vulnerability. Nevertheless, such a shift would be consistent with clinical guidelines recommending surgical inpatient treatment particularly in cases involving organ damage or infertility, which are often associated with longer hospital stays [4, 26, 27]. Although information on one-day hospital admissions and length of stay is available in the underlying hospital statistics, analyses of hospitalization duration or differences between short and longer stays were beyond the scope of the present study, which focuses on population-level hospitalization trends. However, it must also be noted that comparability is certainly limited by the fact that our analysis is based on discharge diagnoses, whereas the studies by Von Theobald et al. (2016) [17], Quesada et al. (2023) [20] and Estes et al. (2019) [24] refer to admission diagnoses.

### Regional differences and potential explanations

The analysis at the federal state level reveals a highly heterogeneous picture regarding endometriosis-related hospitalizations in Germany. While states such as North Rhine-Westphalia, Bavaria, and Saarland reported consistently high hospitalization rates, others, particularly Hamburg, Bremen, and Saxony, showed substantially lower figures. The long-term trends since the year 2000 also vary significantly between federal states. Berlin, Brandenburg, and Bavaria recorded the most substantial increases over time, with Berlin showing the most pronounced rise: between 2000 and 2023, its hospitalization rate increased by 196%, nearly tripling within two decades. At the same time, federal states with comparatively high baseline hospitalization rates, such as North Rhine–Westphalia and Saarland, showed more moderate relative increases over time, which may partly reflect ceiling effects. Regions with already elevated age-standardized hospitalization rates at the beginning of the observation period have less room for further relative increases, whereas states with lower initial rates may show larger relative changes even if absolute levels remain lower. The study by Kohring et al. (2024) also shows considerable differences in the observed crude diagnosis prevalence between the different regions [13]. In contrast, Hamburg and Saxony-Anhalt experienced only moderate growth. Hamburg stood out in particular, not only as the state with the lowest hospitalization rate for endometriosis in 2023, but also for its overall increase remaining well below the national average.

It remains unclear to what extent specific factors might explain the development observed in Hamburg. A particularly striking aspect is that similarly unusual trends can also be observed in the neighboring region of Bremen. However, the prevalence of endometriosis diagnoses has also increased over the years in Hamburg and Bremen, in line with the national trend [13]. In light of these findings, it appears likely that regional differences in hospital admissions are not primarily driven by differences in diagnostic activity. Other factors, such as variations in treatment practices, outpatient care structures, or hospital admission policies, may therefore play a more significant role. Some health care providers may prioritize conservative or outpatient management, while others may more readily admit patients for diagnostic laparoscopy or surgical excision, even in less severe cases. These institutional practices are often shaped by historical treatment paradigms and the availability of gynecologic surgical infrastructure [27]. In regions with lower case numbers, more efficient – or at least more prominent – outpatient care models may contribute to this pattern. However, underdiagnosis, inadequate care, or limited access to specialized treatment centers should also be considered as potential contributing factors [18, 24]. Our findings do not indicate that regions with certified endometriosis centers can account for these developments. However, proximity to such centers could still affect patient referral patterns and access to advanced care [28]. At present, there is a lack of reliable data to assess these relationships in a differentiated manner. Particularly in regions with low hospitalization rates, it is necessary to examine more closely whether this is due to available outpatient care structures or to inadequate diagnosis and treatment. One potential hypothesis is that urban regions such as Hamburg and Bremen may have invested more in outpatient gynecological services, including specialized pain clinics or fertility counseling centers, which could reduce the need for inpatient interventions [20]. Conversely, rural areas might see higher hospitalization rates due to limited outpatient resources [29].

It should also be noted that coding practices and documentation quality can differ between hospital administrations, which may affect the recording of diagnoses and therefore influence observed case rates. Limitations in outpatient billing for complex endometriosis care have been noted, which could either increase or reduce hospital admission rates depending on the region [13].

In addition, cultural and socioeconomic factors may affect health-seeking behavior. Women in certain regions may be more likely to advocate for themselves in the healthcare system or may have better access to health literacy resources, thereby increasing their chances of being referred for surgical intervention [30, 31]. Conversely, in areas with greater social deprivation, barriers to specialist care—such as long waiting times, lack of transportation, or competing health and family priorities—may contribute to the underutilization of hospital-based services. Migration status and language barriers may also play a role, as women with a history of migration might face challenges in accessing or navigating the healthcare system [32]. Regional demographic profiles, including the proportion of foreign-born residents or the prevalence of non-German-speaking populations, could thus influence hospitalization patterns. Although linking hospitalization trends with socioeconomic indicators such as deprivation indices could provide valuable explanatory insights, such analyses were beyond the scope of the present study due to the aggregated nature of the hospital statistics used.

Despite the overall rise in hospitalizations, the wide divergence in case rates therefore suggests persistent inequalities in the diagnosis and treatment of endometriosis across Germany’s federal states. This calls for a more detailed examination of healthcare accessibility, regional care models, and patient pathways. National health reporting systems should aim to include more detailed indicators of outpatient care availability, time to diagnosis, and patient satisfaction to better understand these regional disparities. Future studies using linked or claims-based data, such as health insurance claims from the Research Data Center (FDZ Gesundheit), could substantially extend the present analyses by enabling assessments of patient pathways, outpatient care utilization, treatment patterns, and socioeconomic determinants. Without such efforts, there is a risk that structural inequities in endometriosis care will remain unaddressed, potentially exacerbating reproductive health inequalities for affected women.

### Strengths and limitations

A key strength of this study lies in the use of a consistent, population-based dataset covering all 16 German federal states over more than two decades. To the best of our knowledge, for the first time, nationwide, cross-insurance data on the frequency, distribution, and temporal trends of hospitalizations related to endometriosis in Germany are reported. With a reporting period spanning 24 years, this study addresses a significant gap in the existing research. Age-standardized hospitalization rates (ASHRs) and joinpoint regression (including AAPC) offer robust, comparable insights into temporal and regional dynamics. In addition, the analyses enable a detailed depiction of regional differences. The results of this study underscore the ongoing need to improve and standardize the care of women with endometriosis in Germany. The regional differences identified emphasize the role of state policy and the need for targeted planning and resource allocation at the state level ensuring equitable access to diagnosis and care.

However, the data is subject to specific limitations that must be considered when interpreting the findings. On the one hand, there is an inadequate representation of the reality of healthcare provision. The dataset lacks important clinical details, such as information on disease severity, the type of treatment received (e.g., surgical versus medical), and patient-level sociodemographic variables and comorbidities. Future studies could address this limitation by linking hospital discharge statistics with clinical registries or health insurance claims data, which would allow for more detailed analyses of disease severity, treatment pathways, and patient characteristics. To determine the exact causes of the developments and regional differences observed in this study, further analyses of detailed clinical data, treatment patterns, and patient-specific factors are required. Furthermore, repeated hospital admissions of the same patient are counted as individual cases, which can lead to an overestimation of hospitalization rates. These pieces of information are crucial for realistically assessing the quality of care and treatment needs, evaluating individual therapy outcomes, and identifying gaps in healthcare provision. Longitudinal, patient-level data would be required to distinguish between first admissions and repeated hospitalizations and to assess recurrence and care intensity more accurately. In addition, regional differences regarding standards of care should be examined. Such differences may be influenced by variations in hospital density, the availability of specialized centers, or reimbursement structures; none of which are captured in the available data. It is particularly important to note that a substantial share of endometriosis-related care is provided in an outpatient setting [4, 13]. Combining hospitalization statistics with data on regional healthcare infrastructure and outpatient care provision could help to better explain observed regional patterns and reduce uncertainty regarding access to care.

Finally, the lack of reliable data on both inpatient and outpatient care, including reasons for hospitalization, may result in an underestimation of the actual resource requirements. This, in turn, hinders evidence-based planning and weakens the case for a needs-based expansion of appropriate care structures. Moreover, comprehensive statistics on the frequency and types of surgical interventions for endometriosis in Germany are still lacking. Future research based on integrated inpatient–outpatient datasets and standardized national reporting frameworks could substantially improve needs assessments, support health policy planning, and strengthen monitoring of endometriosis care in Germany.

## Conclusion

The persistent and in some cases widening regional disparities in endometriosis-related hospitalizations likely reflect a multifaceted interaction of factors, including outpatient care infrastructure, hospital admission policies, regional sociodemographic profiles, local medical cultures, diagnostic routines, and structural elements such as funding mechanisms and coding practices. These patterns may be further shaped by variations in disease awareness, access to care, and help-seeking behavior among both patients and healthcare providers. Given the current limitations of available data, a comprehensive understanding of these dynamics remains elusive, underscoring the urgent need for future studies that combine quantitative analyses of healthcare utilization with qualitative insights into patient and provider experiences. 

## Data Availability

Data used in the present study can be obtained from the Federal Statistical Office (http:/www.gbe-bund.de) free of charge.

## Abbreviations

AAPC: Average Annual Percent Change
ASHR: Age-standardized hospitalization rate

## Footnotes

1 The database uses dynamics links which is why no URL for the specific data can be provided here. However, the respective data can be found using the following path: www.gbe-bund.de > DATA SOURCES > Data Sources of the Federal Statistical Office > Hospital statistics - diagnostic data of the hospital patients > Diagnostic data of the hospitals by place of residence (ICD10-3-digits, from 2000).
